# Rock-Boring Ephemera

**Published:** 1881-04

**Authors:** 


### ROCK-BORING EPHEMERA.

At the meeting of the New York Academy of Sciences, April 11, Dr. Trimble of New Jersey, exhibited specimens of marine shells and marble which were deeply perforated by larva of certain ephemera. The marble had been bored in every direction to the depth of from two to three inches, and thus honey-combed with slender passages plugged at the entrance with a closely cemented deposit. In their flying state the ephemera (commonly called May flies or day flies) live but a few hours. The larvæ live in water for a year or more, and, according to Dr. Trimble, secrete an acid which enables them to bore into limestone, passing through their first transformation in the closed burrows.—*Scientific American.*

